# Dr. Crawford W. Long and Anesthesia

**Published:** 1897-10

**Authors:** 


### DR. CRAWFORD W. LONG AND ANESTHESIA.

The claim of Dr. Crawford W. Long to the honor of the first discovery of anesthesia by ether finds another able and earnest defender in Dr. Hugh H. Young, Assistant Resident Surgeon at the Johns Hopkins Hospital, Baltimore. (*Johns Hopkins Hospital Bulletin*, August-September.) The paper reviews the leading well authenticated facts about Dr. Long's use of ether as an anesthetic from 1842 to 1845, and some of the original documents which Dr. Long had collected are again presented; such as the certificate from the first patient operated upon, and another from an eye-witness. Dr. Long's paper before the State Medical Society (Savannah, April 13, 1853), which was in substance the same as his original article in the *Southern Medical and Surgical*

*Journal* (December, 1849), is also published. A portion of the original manuscript of this paper is reproduced in *fac simile.*

Dr. Young does not believe the Wilhite claim. Very properly he regards this claim as without foundation in fact.

The paper of Dr. Young deserves to be widely read; it is a splendid account of Dr. Long's work and an important contribution to the evidence which fully establishes the fact that Dr. Long was the first to discover and practice anesthesia.

